# Recent Advances in Research on the Synthetic Fiber Based Silica Aerogel Nanocomposites

**DOI:** 10.3390/nano7020044

**Published:** 2017-02-16

**Authors:** Agnieszka Ślosarczyk

**Affiliations:** Poznan University of Technology, Institute of Structural Engineering, Piotrowo 5 street, 60-965 Poznań, Poland; agnieszka.slosarczyk@put.poznan.pl; Tel.: +48-61-665-2166

**Keywords:** silica aerogel nanocomposites, sol-gel synthesis, ambient pressure drying, supercritical drying, fibers reinforcement

## Abstract

The presented paper contains a brief review on the synthesis and characterization of silica aerogels and its nanocomposites with nanofibers and fibers based on a literature study over the past twenty years and my own research. Particular attention is focused on carbon fiber-based silica aerogel nanocomposites. Silica aerogel is brittle in nature, therefore, it is necessary to improve this drawback, e.g., by polymer modification or fiber additives. Nevertheless, there are very few articles in the literature devoted to the synthesis of silica aerogel/fiber nanocomposites, especially those focusing on carbon fibers and nanofibers. Carbon fibers are very interesting materials, namely due to their special properties: high conductivity, high mechanical properties in relation to very low bulk densities, high thermal stability, and chemical resistance in the silica aerogel matrix, which can help enhance silica aerogel applications in the future.

## 1. Introduction

**Figure 1:**
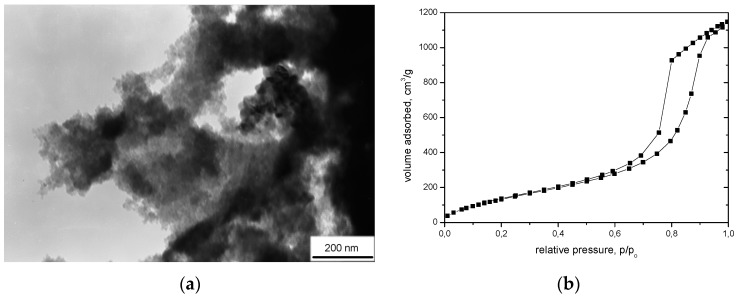
(**a**) Transmission electron microscopy (TEM) picture of silica aerogel from Tetraethyloorthosilane (TEOS) supercritically dried in CO_2_; (**b**) adsorption/desorption isotherms of N_2_ for silica aerogel (curve shape corresponds to the characteristic mesoporous structure).

**Figure 2:**
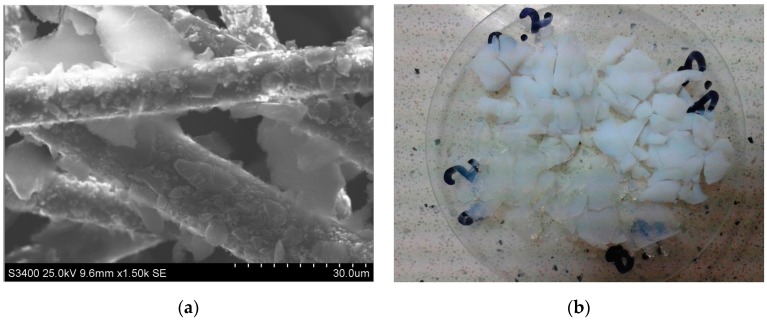
(**a**) Scanning electron microscopy (SEM) picture of silica aerogel blanket; (**b**) Silica aerogel granules.

Silica aerogel consists of small spherical nanoparticles with a size of 1–2 nm which agglomerate to form larger particles with diameters of 5–10 nm, and create a pearl necklace-like structure containing large voids with diameters below 50 nm (Figure 1). The above characteristics give silica aerogels unique properties such as: low density, developed specific surface area, high porosity, low thermal conductivity, and transparency, and enable its application in many fields of science: as thermal and acoustic isolators, catalysts and catalyst carriers, gas filtering and storage membranes, or conductive and dielectric materials for applications in electronics [1,2,3]. Due to the low density below 0.5 g/cm^3^, high surface areas up to 1200 m^2^/g, and very low thermal conductivity below 0.02 W/(m∙K), silica aerogels have found applications as isolating materials in the building and clothing industries. They can be used in the form of blankets, granulates, or powders (Figure 2). An example of isolation in the form of blankets are materials produced by ASPEN or CABOT. ASPEN produces silica aerogel blankets from organosilicon compounds and glass fibers by means of supercritical drying in CO_2_, whereas CABOT products are based on water glass granules and polymer fibers and are made by drying at atmospheric pressure. The thermal conductivities of silica blankets are about 0.020 to 0.040 W/(m∙K) and about 0.020 to 0.025 W/(m∙K) respectively for ASPEN and CABOT blankets, depending on their width [4,5]. Silica aerogel in granulate form has also found applications in different window systems such as: aerogel window [6,7,8] or an innovative glazing system named an aerogel glazing unit (AGUs) [9,10]. In the last system, the gap between two glazings is filled with silica aerogel granules. The authors of study [10] showed that the dimensions of granules play an important role in the optical and thermal properties of such a system. They measured U-value, visible light transmittance, and solar factor for granules with two diameters, 0.5 and 3–5 mm. In the case of small aerogel granules, these parameters were 1.05 W/(m^2^·K), 0.15, and 0.27, respectively, while for larger granules the values were higher, at 1.19 W/(m^2^·K), 0.5, and about 0.57, respectively.

Due to their characteristic mesoporous structure, well-developed specific surface area, and high porosity, silica aerogels have also found applications as catalysts and catalyst carriers. It was shown in many articles that the specific nanostructure of silica aerogels enables the uniform distribution of metal nanoparticles and gives the nanomaterial homogeneous properties in all three dimensions. The obtained nanomaterial is characterized by high porosity coming from the silica aerogel and magnetic, optical, or electrical properties from the metallic nanoparticles. In such a way, the following metallic or bimetalic systems were obtained: Fe/SiO_2_ [11], Ag/SiO_2_ [12], Au/SiO_2_ [13,14], Cu/SiO_2_ [15], Ni/SiO_2_ [16], Fe_2_O_3_/SiO_2_ [17,18,19], TiO_2_/SiO_2_ [20], SnO_2_/SiO_2_ [21], Fe/Mo/SiO_2_ [22], and Fe/Co/SiO_2_ [23]. The disadvantage of such a solution is an increase of the nanocomposite weight. Silica aerogel based catalysts can be applied in many chemical syntheses, such as: the synthesis of nitriles with hydrocarbons and the use of nitrogen oxide II; the conversion of isobutene, with nitrogen oxide on the zinc oxide/aerogel carrier, to methacrylonitrile; or the synthesis of methanol from carbon monoxide on the Cu-Zr/aerogel carrier.

Very good absorption properties of silica aerogels, that result from their specific structures, enable their use in the production of different types of absorbents: for gas CO_2_ as membrane contactors [24], or for particulates in Cherenkov radiation detectors [25]. They can be also used as adsorbents for different organic media. The authors of study [26] used hydrophobic and hydrophilic silica aerogels from water glass as adsorbents for insoluble and water soluble organic compounds, such as rhodamine B and dieldrin. There are also known works where silica aerogels were applied as supports for oral delivery systems, e.g., drugs [27,28]. For example, Sminova and co-workers loaded the silica aerogel with different drugs by adsorption from their solutions in supercritical CO_2_. Thanks to good biocompatibility and high surface areas in the proposed solutions, the drugs supported on hydrophobic silica aerogels dissolved faster than the corresponding crystalline drugs.

## 2. Synthesis of Silica Aerogels

Silica aerogels are usually made in a three-step process consisting of sol-gel synthesis, aging, and drying of the gel. Precursors of aerogels are usually organosilicon compounds (mostly tetramethyloorthosilane TMOS, tetraethyloorthosilane TEOS, polyethoxydisiloxanes PEDS) [29,30,31] or much cheaper sodium silicate (water glass) [32,33]. Cases of synthesizing silica aerogels from other precursors containing silica, such as ash rice [34], bagasse ash [35], fly ash [36], or oil shale ash [37,38] are also known.

Gel is formed as a result of the sol-gel process, where the sol is defined as a colloidal system in the liquid phase, where dispersed solid particles have dimensions from 1 to 1000 nm. In the case of organosilicon compounds, first the silicon precursor is dissolved in alcohol solution, and then the acidic or alkaline catalyst is added. Within a few minutes the gel is created due to the hydrolysis and condensation reactions. Silica gel can be also prepared from water glass in two ways, directly from the water solution of the water glass and from siliceous acid followed by titration of the water glass solution via ion-exchange resin bed. Following this stage of aerogel synthesis is the aging, which aims to dispose of water from the pores of the formed gel. This process can last from several hours to a few days in room temperature or in temperature raised to 50–60 °C, which contributes to a significant reduction of this stage [39,40]. Aging of the gel can be conducted with the use of alcohol applied for synthesis, for example methanol or ethanol, however, the latest research indicates that better parameters of the synthesized aerogel are obtained in the case of applications of higher alcohol homologues, such as *n*-Propanol, *n*-Butanol, Isobutanol, or *n*-Pentanol [41]. The application of alcohol with longer hydroxide chains as solvents resulted in reduction of the volume contraction in the formed aerogel. Moreover, the aging process is important in terms of obtaining a homogenous and transparent silica aerogel in the next synthesis stage, supercritical drying, most frequently in low-temperature processes in CO_2_ or by drying in ambient pressure. Drying in lowered temperature and in lowered pressure is accompanied by high capillary forces, causing contraction and fracture of the gel, as a result of which a xerogel is formed. Most often in order to eliminate these, the so-called Hunt process is applied, where pores are rinsed with CO_2_ in the supercritical phase. The supercritical phase is achieved at the moment of crossing the critical point of a given substance, by obtaining the appropriate temperature and pressure (for CO_2_ it is 31.1 °C with a pressure of 73.7 bar). During gel drying the solution is removed from the pores of the synthesized gel and the structure is filled with air only, which defines the specific properties of the nanomaterial obtained [42,43,44]. This step can be also performed in a high-temperature process, usually in alcohol, but the critical parameters of the process are considerable higher, e.g., for methanol and ethanol T_cr_ = 239.4 °C, P_cr_ = 8.09 MPa, and 243.2 °C, 6.38 MPa, respectively [45,46]. Due to high cost of this type of drying and the explosion danger, along with the studies on supercritical drying, research on forming silica aerogel dried in conditions of atmospheric pressure were conducted. Here a modification of the gel called silylation is performed before drying where the hydroxide groups –OH localized on the silica gel frame are replaced by inert hydrocarbon groups –O–Si(CH_3_) [47,48,49,50]. Most commonly this modification is made with the use of trimethylchlorosilane (TMCS), hexamethyldisiloxane (HMDSO), hexamethyldisilazane (HMDZ), methyltrietoxysilane (MTES), or methyltrimethoxysilane (MTMS). The concept of this method is to give the structure of the silica aerogel a hydrophobic character, so that further hydrolysis of the formed aerogel is impossible, and neither is its volume contraction causing its contraction and transformation into a xerogel. A disadvantage of this modification method is that it is time consuming. Forming a hydrophobic aerogel takes place gradually, by repeated exchange of the solvent. This process can last from several hours to a few days depending on the applied temperature. Thus, in recent years there have been works concerning the formation of silica aerogels in a one stage process with the use of drying in atmospheric pressure. Researchers synthesized the silica gel using TEOS and TMCS compounds as precursors. As a result they formed a hydrophobic silica aerogel with a much shorter synthesis time and with less solvents applied, which made the whole process significantly cheaper [51].

## 3. Fiber-Based Silica Aerogel Nanocomposites

**Figure 3:**
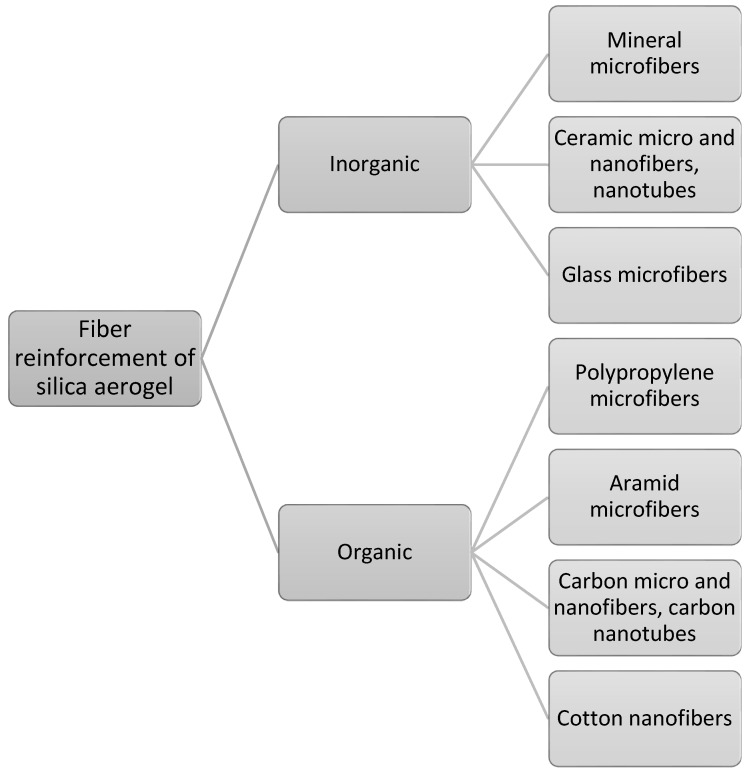
Scheme of different fibers used as reinforcements in silica aerogel nanocomposites.

The wide application of silica aerogels on an industrial scale is limited by the brittleness of this material. Reinforcement of the silica aerogel structure can be introduced via different methods; the inbuilding of the polymer chains or reinforcing the structure with dispersed fibers or fiber mats being the most common. The simplest way to synthesize these type of composites is by the introduction of short fibers to the solution of the silicon precursor before gelation. A significant stage of the synthesis is the correct timing of gelation, so that the homogeneous structure of the composite is obtained and sedimentation of the fibers does not occur. In such a way, the different types of fibers based on inorganic or organic precursors were introduced in the silica aerogel matrix (Figure 3).

### 3.1. Silica Aerogel Nanocomposites with Inorganic Microfibers

The first research on the synthesis of nanocomposites from silica aerogels with the addition of short fibers appeared in the 1990s [52]. In the initial stage of the research, ceramic microfibers were mostly applied as the reinforcement of the silica aerogel structure, because of their high heat resistance corresponding to the high heat resistance of the silica frame. Parmenter and Milstein formed a silica aerogel from TMOS with a mixture of ceramic fibers 1.27 cm long with amounts ranging from of 5 to 25 wt % (silica fibers 68%, alumina fibers 20%, and aluminaborosilicate fibers 12%, with diameters of 3, 2–4, and 8 µm, respectively) with supercritical drying in a methanol solution. However, the results of the addition of those fibers were not as spectacular as was expected. Due to a relatively high specific density, the ceramic fibers significantly increased the density of the nanocomposites, from 240 kg/m^3^ for pure aerogel to 330 kg/m^3^ for aerogel with a 10% fiber addition, and decreased its mechanical parameters, such as compressive strength, hardness, and elastic modulus. For pure aerogel, the compressive strength was about 1 MPa, whereas with the fiber addition it oscillated from 0.34 to 0.79 MPa. Mechanical parameters were improved by about 20%–40% with the smaller share of fiber additions, about 5–10 wt %. Those fibers, added in higher amounts (25 wt %), positively influenced the contraction of the silica frame during supercritical drying. Much better parameters of the composites from silica aerogels reinforced with ceramic fibers were gained in 1998 by Deng and in 2013 by Yang. The first, in order to reinforce the silica frame, used ceramic microfibers that were 70 µm long 10 µm in diameter in the amount of 10 wt % [53]. Additionally, they used kaolinite and attapulgite for reinforcing the structure of the silica aerogel. The sol-gel synthesis was conducted using TEOS in the solution of ethanol and in the presence of the acidic catalyst HF. Drying of the nanocomposite was conducted in supercritical conditions in ethanol. Research proved that the presence of the ceramic fibers and mineral fillers significantly improved the compressive strength from 1.8 × 10^4^ Pa to 12.8 × 10^4^ Pa, and the density of the nanocomposite from 0.112 g/cm^3^ to 0.185 g/cm^3^, respectively, for pure aerogel and the aerogel with additions. Yang, on the other hand, used longer ceramic fibers (30–40 mm long and 4–5 µm in diameter in the amount of 7% of the volume) to reinforce the silica frame [54,55]. However, in this case, another way of forming the nanocomposite was proposed. In the first step, the fibers were formed via pressing the material between steel covers, and then they were impregnated with initially hydrolyzed sol from TEOS and the reaction catalyst (ammonium hydroxide) was introduced. After gelation, the process of drying was conducted in supercritical conditions in an ethanol solution. The composite formed in this way was characterized by a density of about 0.29 g/cm^3^. The researchers tested the relation between stress and strain during compression within the temperature range from 25 to 600 °C in the perpendicular direction of the load. They noticed that the stress-strain curves in all the tested temperatures had the same destruction characteristics, which can be divided into three stages: the linear stage, the yielding stage, and the densification stage. Wang accredits those stages with the following mechanisms: in the first elastic stage, the load is carried by the silica frame; in the second—inelastic stage, after the matrix cracks, the load is carried by the fibers; and the third stage depends on the degree of the aerogel structure density, the strength of the fibers, and the adhesion of the fibers to the silica matrix [56]. In the first and the second stage, up to a strain of 0.3 mm/mm, there was no significant influence of temperature on the compressive strength of the composite. Only with a strain over 0.5 mm/mm was a significant rise of the compression stress observed together with the temperature rise. The formed composites had higher compressive strength and deformability than the earlier tested pure silica aerogels. The compressive strength in temperatures of 25, 400, 600, and 800 °C were respectively: 13.5, 14.9, 19.2, and 25.8 MPa. This specific conduct of the composite in higher temperatures was attributed by the authors to the formation of large clusters of aerogel nanoparticles, fiber stress relaxation, and gradual cracking and moving of the fibers from the aerogel structure. Very good mechanical parameters of the composites based on the silica aerogels were also gained when the mullite fibers were used as the silica frame reinforcement. Li and Su used mullite microfibers that were 100 to 400 µm long and 2 to 15 µm in diameter [57]. To prepare a composite with higher thermal resistance, the sol-gel synthesis was conducted with the use of two precursors: silicon precursor TEOS and zirconium precursor ZrOCl_2_. The synthesis was conducted with the use of the acidic catalyst HNO_3_ and epoxides as the accelerator of gelation; whereas the drying was conducted in supercritical conditions in ethanol, in the high-temperature process. Mullite fibers were dosed from 4 to 10 vol %. Research proved that the higher volume amount of fibers increased the density of the aerogel nanocomposite from 0.225 g/cm^3^ for pure aerogel to 0.419 g/cm^3^ for nanocomposites with a 10% amount of fibers. In the case of the highest content of fibers, the highest value of compressive strength was observed—0.438 MPa, which was about 35% higher than the value gained for pure silica aerogel. Moreover, research proved that the presence of such high amounts of fibers in the structure of the aerogel, despite the higher density of the nanocomposite, did not change the characteristic mesoporous structure of the composite, while the high porosity of the structure at 90% and very good insulating parameters (thermal conductivity equaled about 0.027 W/(m∙K)) were maintained. The cause for such good insulating parameters of the nanocomposite, according to the authors and the Ryu research [58], is the specific structure where thanks to rapid gelation, the fibers are divided by a layer of aerogel, which keeps them from crossing and causing an increase of thermal conductivity via creation of fiber-to-fiber connections. Another solution, offered by Kim and his co-workers, is an aerogel composite reinforced with short glass fibers [59]. Contrary to earlier solutions, drying of the aerogel composite took place in atmospheric conditions. The authors synthesized the silica xerogels from TEOS and colloidal silica in various volumes (0, 25, 50, 75, and 100%) using as the reinforcement the fiberglass and the chemical modification of the gel surface in the TMCS/*n*-hexane mixture. The best parameters were gained for a xerogel from a mixture of TEOS and colloidal silica; the density of the composites oscillated around 0.116 to 0.143 g/cm^3^, and specific surface areas from 397 to 456 m^2^/g.

### 3.2. Silica Aerogel Nanocomposites with Organic Microfibers

Reinforcing the silica aerogel structure can be also obtained by the application of organic fibers. Zhang and his co-workers applied aramid fibers 10 cm long and with 13 µm diameter in the amount of 1.58 to 11.4 wt % as the silica aerogel frame [60,61]. Despite the lack of chemical reaction between the fiber surface and the silica matrix, with an increasing amount of fibers, an increase of the flexural strength of the composite was obtained, from 0.025 to 0.18 MPa, as well as an increase in the thermal conductivity from 0.022 to 0.025 W/(m∙K). An increasing amount of aramid fibers made the specific surface area of the composite shrink, lowered its density, and significantly lowered the thermal resistance of the composite, in comparison with pure silica aerogel. On the other hand, the researchers of study [62] used polypropylene fibers that were 12 mm long as the reinforcement of the silica aerogel structure. Silica xerogels formed in this way were characterized by very good structural parameters (specific surface areas around 700 m^2^/g and mesoporous structures with three types of pores: cylindrical with both ends open, cylindrical with one end closed or with narrow neck). Moreover, the formed composite had a much higher adsorption capacity than activated carbon fibers, or granules of activated carbons, and it had a higher mechanical resistance than pure silica aerogel. An interesting solution is the application of cotton fibers as the silica xerogel frame. The authors of the study [63] formed silica xerogels from water glass with a high amount of cotton fibers oscillating from 20 to 80 wt %. The research proved that by increasing the fiber amount, the density of the composite lowered from 0.140 for pure aerogel to 0.078 g/cm^3^ for the aerogel with 80% fiber content, whereas the thermal conductivity of the composite increased from 0.013 to 0.025 W/(m∙K). A side effect of adding this amount of hydrophilic fibers was the decrease of the contact angle of the formed composites.

### 3.3. Silica Aerogel Nanocomposites with Inorganic and Organic Nanofibers

Very good parameters of silica aerogel nanocomposites were obtained when nanofibers were applied as the reinforcement. The authors of study [64] applied, as the reinforcement of the silica frame, sepiolite fibers of 2–10 µm length with hydrated magnesium silicate with the formula Si_12_Mg_8_O_30_(OH)_4_(OH_2_)_4_·8H_2_O and specific needle like structures. As a result of addition of the sepiolite fibers (from 0.5 to 1.5 vol %) to the silica matrix, nanocomposites were formed with a density value close to the pure aerogel—about 0.20 g/cm^3^ and with much less contraction during drying. In the case of pure aerogels the contraction was over 17%, and its value was lowered together with the increase of the fiber amount, reaching a value of 4.3% in the case of the highest fiber volume. Moreover, the addition of the sepiolite fibers increased the compressive strength of the silica aerogel from 0.1 to 1.1 MPa with the highest fiber volume, and slightly increased the thermal conductivity of the aerogels from 0.021 to 0.026 W/(m∙K). A negative effect of the addition of the sepiolite nanofibers was the reduction of the specific surface area of the silica aerogels from 1.039 to 795 m^2^/g. Despite this, the analysis of the adsorption/desorption isotherms N_2_ showed no influence of the nanofibers on the structure of the formed materials; both in the case of the pure aerogel and nanocomposites with sepiolite fibers, a mesoporous structure with open cylindrical pores was obtained. A similar effect of the reduction of the specific surface area of the aerogel after addition of polyaniline nanofibers was also observed [65]. Loy and his co-workers showed that the introduction of the nanofibers reduced the specific surface area of the aerogel from 1330 m^2^/g to 927 m^2^/g for the composite with 16.5 wt % of nanofibers, and the reduction was relatively small in comparison with the aerogels modified with polymers, where the specific surface area was reduced usually by 75%. Moreover, the authors of the study proved that the bending strength of the composite reinforced with polyaniline nanofibers increased linearly together with the addition of more and more fibers, up to a composite density of 0.074 g/cm^3^, which corresponded with a 3 wt % amount of nanofibers, and then it dropped, but for all the amounts of the nanofibers an improvement of the composite strength was observed in comparison with the pure aerogel. The strength of a composite with a 3% addition of nanofibers was about 200 times higher than the case of the pure aerogel. It was also demonstrated that the addition of polyaniline nanofibers improved the electrical conductivity of the composite. The highest conductivity of 1.83 × 10^−5^ S/cm was registered for the composite with the highest nanofiber addition, 16.5 wt %. Another very interesting example of nanofibers used as silica aerogel reinforcements are silica nanowires [66]. The silica nanowires with lengths of 2, 3, 10, and 20 µm were synthesized in a one-pot method from TEOS solution in the presence of polyvinylpyrrolidone, *n*-penthanol, and sodium citrate aqueous solution, with ammonia as a catalyst. The silica aerogel nanocomposite was formed as follows: the silica nanowires were added to the ethanol solution, and in the next step the TEOS solution was added. The mixture was first hydrolyzed by adding hydrochloric acid, and then the gel was formed in the presence of the ammonia catalyst. In the next stage, the gel was modified in TMCS/ethanol/*n*-hexane solution at 35 °C and dried in air. The authors of the study showed that the silica wires used in the amounts of 3.5 to 14 wt % decreased the specific surface area of the pure silica aerogel negligibly from 950 to 847 m^2^/g and increased the density of the nanocomposite from 0.151 to 0.193 g/cm^3^. They obtained a good dispersion of silica wires in silica sol due to good compatibility of both components. Moreover, the presence of silica wires in silica aerogel increased the thermal conductivity of the nanocomposite (from 0.021 to 0.027 W/(m∙K)), and improved the compressive strength, which increased from 0.3 to 3.5 MPa.

### 3.4. Silica Aerogel Nanocomposites with Inorganic Nanotubes

A very distinctive solution is the application of inorganic nanotubes as silica aerogel reinforcements. The first example is of tungsten disulfide nanotubes, characterized by very good structural and mechanical parameters. The Young’s modulus is about 150 GPa, and the tensile strength is 16–20 GPa. They have very good thermal stability up to 350 °C in an oxygen atmosphere and almost 1000 °C in an inert atmosphere. Similar to multiwall carbon nanotubes, the tungsten disulfide nanotubes are composed of layers joined by van-der-Waals forces, while the outer surface consists of inert sulfur atoms. The specific structure leads to very weak intermolecular forces between WS_2_ nanotubes and enables their good dispersion in organic matrices, from which the most often tested were polymers. Due to their very interesting properties, the authors of study [67] decided to use WS_2_ nanotubes in the silica aerogel matrix. WS_2_ nanotubes were introduced to the catalyst solution, sonicated, and then the TEOS in ethanol solution was added. The gelation took place after 20–25 min. The drying stage was performed by means of CO_2_ under supercritical conditions. Bar and coworkers showed that tungsten disulfide nanotubes in the amount of 0.1 wt % increased the structural parameters of silica aerogel; the specific surface area value rose from 848.4 for pure silica aerogel to 857.4 m^2^/g for the nanocomposite. Moreover, WS_2_ nanotubes improved the mechanical properties of the nanocomposite; the apparent toughness of the composite increased by about 23%. It was also shown that higher amounts of nanotubes (0.25 wt %) diminished the mechanical properties of the silica aerogel nanocomposite, while lower concentrations (0.05 wt %) did not show any effect. Very good structural and mechanical parameters of silica aerogel nanocomposites were also obtained with three-dimensional halloysite nanotube reinforcement [68]. Liu and coworkers used 0.2–1.5 µm length halloysite nanotubes with a nanotubular structure characterized by an inner diameter of about 10–35 nm, and wall thickness of about 10–20 nm. Halloysite nanotubes are environmentally friendly, have good mechanical and thermal properties, and due to low cost of production can be an inexpensive alternative to more expensive carbon nanotubes. The study showed that halloysite nanotubes in the amounts of 1 to 10 wt % decreased the density and specific surface area of the silica aerogel, while they improved the shrinkage and mechanical parameters of the nanocomposite. The best results were obtained for silica aerogel strengthened with 10 wt % of halloysite nanotubes; the compressive strength was 1.45 MPa, shrinkage was 14%, and the density was only 0.12 g/cm^3^. Additionally, the higher amounts of nanotubes increased the thermal conductivity of the silica aerogel nanocomposite to 0.038 W/(m∙K) in comparison with the pure silica aerogel (0.025 W/(m∙K)).

## 4. Silica Aerogel Nanocomposites with Carbon-Based Fibers in Nano and Microscale

Presentation of the literature on fibers and nanofibers reinforcing the silica frame showed that so far, this had been the best method to improve the resistance parameters of silica aerogels. There are relatively few articles on carbon fiber/silica aerogel composites, despite the fact that carbon fibers, in comparison with glass or ceramic fibers, are characterized by very good mechanical parameters, especially in relation with their density, dimensional stability up to a temperature of 750 °C, low coefficient of thermal expansion, fatigue resistance, biological compatibility, and very good electrical conductivity. Thanks to those properties, carbon fibers are applied in many industries, such as aerospace, automotive, chemical engineering, missile, nuclear field, reinforcement in composite materials, and textiles [69,70].

Despite the good characteristics of carbon fibers and nanofibers, there are relatively few studies on reinforcing the silica aerogels and xerogels with those materials. The first studies on this subject appeared in 2004, when Lu and his co-workers used carbon fibers with diameters of 30–100 nm and length of 100 µm in amounts from 0.5 to 20 wt % as reinforcements for silica aerogels [71]. Research proved that the addition of carbon nanofibers significantly lowered the density and the specific surface area of the aerogel nanocomposite; for a 20% fiber addition, the composite density was 0.110 g/cm^3^, and the specific surface area was 696 m^2^/g; for pure aerogels those values were respectively 0.218 g/cm^3^ and 1199 m^2^/g. Introducing such a large amount of nanofibers into the structure positively influenced the thermal conductivity of the aerogel. Carbon fibers with a very low index of thermal conductivity—0.07 W/(m·K)—lowered the thermal conductivity of pure aerogel from 0.0410 W/(m·K) to 0.0295 and 0.0380 W/(m·K) for 0.5% and 20% fiber addition, respectively. Moreover, it was found that the carbon fibers characterized by high extinction coefficients (3.75 to 5.06) significantly limited the thermal radiation of the aerogel in higher temperatures and lowered the thermal conductivity of the silica aerogel at 500 °C. This phenomenon significantly prolongs the thermal resistance of the aerogel composite and makes it possible to use it as high temperature insulation. Another solution offered by Meador and his co-workers was the application of carbon nanofibers with di-isocyanate [72,73]. To reinforce the silica frame they used carbon nanofibers with a high degree of order and carbon content over 99.9% in amounts from 0 to 5 wt % in relation to the silicon precursor mass. Fibers were introduced into sol modified with APTES amine, and then the solution was cross-linked via the addition of di-isocyanate in amounts from 6 to 34 wt %. It was observed that the introduction of carbon nanofibers in the amount of 5 wt % into the aerogel structure with a density below 0.1 g/cm^3^ significantly improved the mechanical properties of the composite—in the tests, the elastic modulus increased by three times, and the flexural strength by five times. In the case of aerogels with a higher density and with a lower concentration of di-isocyanate, a further increase of the mechanical parameters was observed, though it was not as spectacular as for lighter composites. With the maximum content of di-isocyanate and a high concentration of the silicon precursor, the best mechanical parameters of the composite were gained with a fiber amount of 2 wt %. Higher amounts of fibers caused the elastic modulus and the flexural strength to decrease relatively, from 182.36 and 5.42 MPa (for 2 wt %) to 164.67 and 5.1 MPa (for 5 wt %). Such material behavior was caused by fiber agglomeration and difficulties with their regular dispersion in the solution containing a high concentration of silicon precursor and the cross-linking factor. Despite very good mechanical parameters, those composites were characterized by very small specific surface areas, oscillating between 160 and 217 m^2^/g.

**Figure 4:**
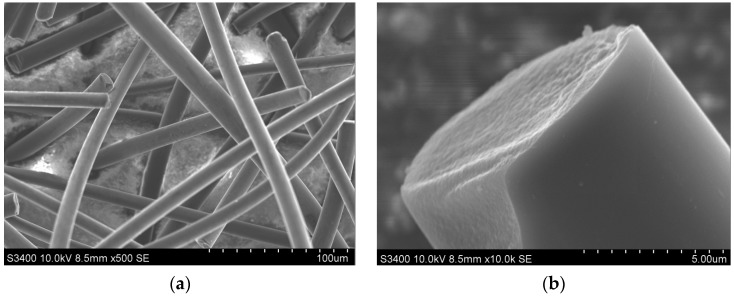
SEM images of carbon microfibers. (**a**) in bulk; (**b**) cross-section of single fiber.

On the other hand, carbon microfibers were applied as the reinforcement of the silica aerogel structure by the authors of [74,75]. A feature that distinguishes this research is the oxidizing modification of the fiber surface before their application in the aerogel nanocomposites. It was indicated that within the oxidative modification of the carbon fibers, the oxygen functional groups are formed on the carbon fiber surface, which in turn, react with hydroxide groups that exist on the silica gel frame and create durable, ionic bonds. As a consequence, the hydroxide groups localized on the silica aerogel are blocked and a more hydrophobic silica aerogel with a higher resistance to shrinkage is created. As reinforcements of the silica aerogel frame, the carbon microfibers from coal-tar pitch with 700 µm length and 13 µm diameter, were produced by DONACARBO (aspect ratio 54, Figure 4). Those fibers are characterized by very good mechanical parameters (tensile strength 750 MPa, elastic modulus 40 GPa), high thermal stability up to 750 °C, density of 1.64 g/cm^3^, carbon content over 99%, and a relatively high degree of structural order. The distance between the graphite planes defined in the Bragg equation was about 0.353 nm. Naturally those fibers are characterized by a low degree of specific surface area development, around 4 m^2^/g, and high hydrophobicity. Thus, the fibers applied in the aerogel composites were first chemically modified in concentrated nitric acid, at higher temperature in order to oxidize the fiber surface and give it a hydrophilic character, which was described in detail in previous articles [74,75].

In the first step of silica aerogel nanocomposite preparation, the carbon microfibers were added to the organosilicon precursor (TEOS) in ethanol solution and mixed for a few minutes in order to get a good dispersion of the fibers. After that the reaction catalyst was added, and within a few minutes the gel was formed. Then the samples were aged for 24 h in a water solution of ethanol, and then for 7 days in pure ethanol. The next step was the modification of the silica gel/carbon microfiber composite in a mixture of TMCS/*n*-hexane in a volumetric ratio of 1:5 for 48 h at a temperature of 50 °C. In the following stage, the gels were placed for 24 h in *n*-hexane solution, and then dried at atmospheric pressure. In Table 1, the example results of the structural parameters formed for the silica aerogel and its nanocomposite with unmodified and modified carbon microfibers are presented.

**Figure 5:**
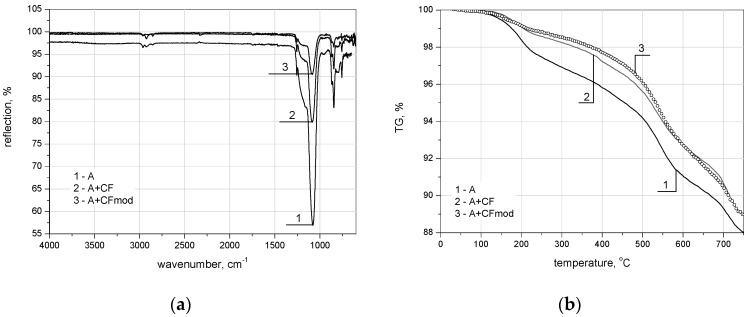
(**a**) Fourier transform infrared spectroscopy (FTIR) and (**b**) TG curves of carbon fiber-based silica aerogels synthesized from TEOS precursor in ambient pressure drying (1—pure aerogel, 2—with unmodified carbon microfibers, 3—with modified carbon microfibers).

The research shows that the carbon microfibers lowered the structural parameters of the formed nanocomposites significantly. In the case of the nanocomposite with unmodified fibers, the specific surface area and the average pore diameter equaled 820.9 g/cm^3^ and 10.9 nm, and 775.1 g/cm^3^ and 10.7 nm for the nanocomposite with modified fibers, respectively. The adsorption/desorption isotherms N_2_ formed for the unmodified silica aerogel and silica aerogel/carbon fiber nanocomposite were typical for the materials with mesoporous structures. Moreover, the carbon microfibers resulted in substantially lowering the nanocomposite density and limiting the silica gel shrinkage during drying. Densities of the nanocomposites with unmodified and modified carbon microfibers were 0.13 g/cm^3^ and 0.12 g/cm^3^, respectively, and the shrinkage for the pure silica aerogel was 27.7 %, and for its nanocomposites with unmodified and modified carbon microfibers was 10.7% and 8.8%, respectively. The lower shrinkage of the silica aerogel nanocomposite with modified carbon microfibers is the result of the chemical reaction between the functional groups on the fiber surface and the hydroxyl group on the silica aerogel frame, and is confirmed by the lowered intensity of the band in the Fourier transform infrared spectroscopy (FTIR) spectrum, from 1050 to 1100 cm^−1^ corresponding to the Si–O–Si bonding of the silica aerogel frame, presented in Figure 5a. For silica aerogel without and especially with unmodified carbon fibers, the increase of this band was observed. On the pure silica aerogel surface, a higher amount of hydroxyl and hydrocarbon groups occur, and therefore on the FTIR spectrum the increase of the intensity of the band at 1050 to 1100 cm^−1^ takes place [51,76]. This effect is strengthened by the addition of unmodified carbon microfibers which disorganize the silica aerogel structure and introduce the additional free spaces with functional groups containing oxygen.

**Figure 6:**
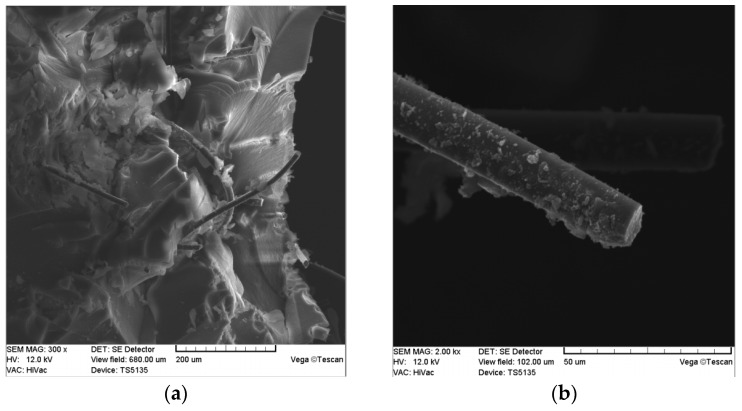
(**a**) SEM pictures of carbon fiber-based silica aerogel; (**b**) the surface of carbon microfibers covered by nanoparticles of the silica aerogel.

The results of the FTIR analysis are comprehensible with the thermogravimetric TG measurements presented in Figure 5b. The analysis proved that the chemical treatment of the silica aerogel/oxidized carbon microfiber nanocomposite in TMCS/*n*-hexane solution provided better thermal resistance in the entire observed temperature range. The confirmation of a good adherence of the oxidized carbon microfibers to the silica aerogel structure is the presence of silica aerogel particles on the fiber surface depicted on the SEM pictures in Figure 6. Probably, the functional groups present on the oxidized carbon microfiber surface are the places from which the silica gel chain increase is initiated. The effect of this is a stable nanostructure. It is also worth mentioning that the obtained structural parameters of the nanocomposites were comparable with the results obtained for composites dried in CO_2_ in supercritical conditions presented in study [75]. The simultaneously modification of silica aerogel by surface treated carbon microfibers and chemical modification in TMCS/*n*-hexane solution produced the hydrophobic structure characterized by low shrinkage and very good structural parameters.

## 5. Conclusions

In this article, the literature study concerning silica aerogel nanocomposites with short microfibers, nanofibers, and nanotubes was presented. This analysis proved that the addition of fibers significantly influenced the resistance of the silica aerogel to cracking, and is one of the best methods to diminish the brittleness of this matrix. The wide range of materials used for the production of fibers allows us to shape and modify the different parameters of the silica aerogels; polymer fibers give a significant increase of the elasticity of the silica matrix, but they lower its resistance to high temperatures, whereas ceramic or glass fibers improve the thermal resistance, but very often increase the density and decrease the specific surface area of the nanocomposite. The application of nanofibers from polyaniline results in forming aerogel nanocomposites with conductive properties, while silica nanowires provide very good compatibility between the silica aerogel matrix and silica oxide based nanowires and leads to very stable nanocomposite structures. Some interesting alternatives are the carbon fibers, nanofibers, and nanotubes, which join the parameters of inorganic and organic fibers. Fibrous-based carbon materials, characterized by good mechanical parameters and relatively low densities, are resistant to temperatures above 600 °C and are biocompatible, which enables their application as components of nanocomposites with silica aerogels not only in the technical industry, but also in the biotechnology field, e.g., as biosensors, diagnostics, or food packaging. Moreover, those fibers can be surface modified, which enables the chemical bonding of functional groups on the fiber surface with hydroxyl groups present on the surface of the silica aerogel, and results in obtaining a nanocomposite with better structural parameters. It is also worth mentioning that there are many research studies in which the silica aerogel nanocomposites are synthesized by ambient pressure drying and the formed nanostructures have very good structural and mechanical properties, comparable with those obtained in supercritical conditions. This leads to a lower cost of the final product and makes possible the use of silica aerogel-based nanocomposites at a larger scale.

## Figures and Tables

**Table 1:** Structural characterization of silica aerogel (1) and silica aerogel/carbon fiber nanocomposite with unmodified (2) and modified (3) carbon microfibers.

Marked in Text	1 (A)	2 (A + CF)	3 (A + CFmod)
Density, g/cm^3^	0.20	0.13	0.12
Surface area by Braunauer-Emmet-Teller (BET), m^2^/g	863.9	820.9	775.1
Average pore diameter, nm	12.2	10.9	10.7
Volume shrinkage, %	27.7	10.7	8.8
